# Everolimus and exemestane in long survival hormone receptor positive male breast cancer: case report

**DOI:** 10.1186/s13104-016-2301-2

**Published:** 2016-11-28

**Authors:** Z. Ballatore, M. Pistelli, N. Battelli, A. Pagliacci, M. De Lisa, R. Berardi, S. Cascinu

**Affiliations:** Clinica di Oncologia Medica e Centro Regionale di Genetica Oncologica, Università Politecnica delle Marche, AOU Ospedali Riuniti-Ancona, Ancona, Italy

**Keywords:** Male breast cancer, Everolimus, Exemestane

## Abstract

**Background:**

Male breast cancer is a rare event, accounting for approximately 1% of all breast carcinomas. Although men with breast cancer had poorer survival when compared with women, data on prognosis principally derive from retrospective studies and from extrapolation of female breast cancer series. We reported the case of a very long survival patient.

**Case presentation:**

A caucasian 42-year-old man underwent radical mastectomy with axillary dissection for breast cancer in 1993. Pathologic stage was pT4pN0M0 infiltrating ductal carcinoma of right breast without lymph nodes metastases. Biological characterization was not available. He received adjuvant treatment with chemotherapy, six cycles of cyclophosphamide, methotrexate and fluorouracil, then endocrine therapy with tamoxifen for 5 years and complementary radiotherapy. Then he began clinical-instrumental follow up. In May 1996, a computed tomography scan showed multiple lung metastases. Hereafter he received several oncologic treatment including seven chemotherapy and five endocrine therapy lines with two re-challenge of endocrine therapy. In October 2007 further lung progression was showed and a biopsy was performed to characterize the disease. Histological examination confirmed breast cancer metastases, immunohistochemistry showed positive staining for estrogen receptor, negative for progesterone receptor and human epithelial growth factor receptor 2, proliferative index was 21%. In April 2013, bone disease progression was evident and he received radiant treatment to sacral spine. In May 2014 an off-label treatment with exemestane and everolimus combination was approved by Ethics Committee of the Marche Region. The patient received treatment for 3 months with evident clinical benefit to subcutaneous lesions of the chest wall that were not visible nor palpable on physical examination after 1 month of treatment.

**Conclusion:**

That is the case of long survival male breast cancer patient with luminal B subtype and no BRCA mutations. He achieved higher progression free survival with endocrine therapy creating the rationale for last line treatment with everolimus and exemestane combination. Attending conclusive results from ongoing studies, everolimus and exemestane should not be used routinely in male metastatic breast cancer patients, but taking into account for selected cases. At the best of our knowledge, this is the first case of male beast cancer treated with exemestane and everolimus combination.

## Background

Male breast cancer (MBC) is a rare event, accounting for approximately 1% of all breast carcinomas and less than 1% of all male cancers [1–3]. Recent data by The International Agency for Research on Cancer, a division of the World Health Organization, demonstrated the global incidence of MBC stands at nearly 8000 cases. For Europe, this equates to 3750 cases of MBC [4]. In 2014 in the United States, 2360 cases were estimated, while the lifetime risk of men getting breast cancer is 1 in 1000 [1]. These figures are significantly lower (<1%) than the incidence of breast cancer (BC) in women, which represents 11.6% of the global cancer incidence in 2014 [5].

Men tend to present 5 years later than women do, commonly in the seventh decade [6, 7]. MBC incidence seems increasing and in the absence of formal epidemiological studies, reasons for the apparent rise could only be speculated [8]. A large population-based study of 2537 men with breast cancer, analyzed from the National Cancer Institute’s Surveillance, Epidemiology and End Results (SEER) database, reported that over a 25 year period (1973–1998) the incidence of MBC increased significantly from 0.86 to 1.08 per 100,000 population in the United States [9].

MBC prognosis is reported to be poorer than female BC one, even if data mainly derived from retrospective studies and from extrapolation of female BC series. Endocrine therapy seems to be effective and many studies report that aromatase inhibitors (AIs) may be an effective and safe treatment option for hormonal receptor (HR) positive, metastatic male breast cancers progressing on tamoxifen [10–14].

Unfortunately, not all patients respond to first-line endocrine therapy (primary or de novo resistance), and even patients who have a response will eventually relapse (acquired resistance).

Everolimus, an mTOR inhibitor, in combination with exemestane demonstrated a 6-month improvement in women with resistance to non-steroidal aromatase inhibitors [15].

In clinical practice, this could be good rationale for the everolimus combination use to overcome the resistance, also in MBC, unfortunately until now there are no data in this setting of patients.

We report the case of a very long survival male breast cancer patient who was affected by hormone-sensitive disease and received an off label combination treatment with everolimus plus exemestane, as a 13th line of treatment, approved by Ethics Committee of the Marche Region (CERM).

According to our country’s legislation, since it was a retrospective study, with no direct patient involvement, the ethical approval and patients consent for the study were not required (Official Gazette No. 72 of March 26, 2012). The medical record was reviewed to find data on patient’s medical history and a written informed consent was obtained from the patient’s next of kin for publication of this Case Report and any accompanying images. A copy of the written consent is available for review by Editor in Chief of this journal.

## Case presentation

We describe the case of a 42-year-old Caucasian man who was diagnosed with BC in 1993. The patient reported a case of BC in his family, anyway without a medical history of risk factors for BC. In June 1993, he underwent to a radical mastectomy with axillary lymphadenectomy; histopathological examination confirmed infiltrating ductal carcinoma of the right breast with poor cellular differentiation, G3, large areas of necrosis, infiltration of subcutaneous tissue and muscular involvement. Biological characterization was not available. The final pathologic stage was pT4pN0(0/12)M0. He was given adjuvant treatment with six cycles of CMF (cyclophosphamide 600 mg/m2, methotrexate 40 mg/m2, fluorouracil 600 mg/m2) administered day 1–8, every 4 weeks, endocrine therapy with tamoxifen 20 mg daily for 5 years and complementary radiotherapy on the chest wall. In May 1996, during follow-up, a computed tomography (CT) scan revealed multiple lung metastases. Hereafter the patient started a first-line weekly paclitaxel chemotherapy, obtaining a partial response. In March 1997, paclitaxel was stopped because of lung metastases progression and an endocrine therapy with megestrol was administered until January 2000 when it was discontinued for a vein thrombosis and switch to exemestane. In January 2001, a new CT showed a further progression of disease (PD). Epirubicin and docetaxel were administered until June 2001 when a further lung progression was revealed. Thus, patient was given letrozole, 2.5 mg daily, until January 2002 when his lung disease progressed. A course of vinorelbine was started but in December 2002 chemotherapy was interrupted for PD and a re-challenge with megestrol produced a partial response until October 2007 when disease progressed again. We performed a lung biopsy to confirm etiology and histological examination was positive for BC metastases, immunohistochemistry showed positive staining for the estrogen receptor (ER) 99%, negative for progesterone receptor (PR) 0, 1% and for herceptest (1+). Ki67 was 21%. In October 2007, a re-challenge was done with tamoxifen, previously used in the adjuvant setting, and in May 2008, with a stable disease and a poor compliance, it was suspended. In January 2009, lung metastases progressed and capecitabine was prescribed observing stable disease for 2 years.

From February 2011 until March 2013, a metronomic combination of cyclophosphamide and methotrexate was administered. In April 2013, bone and nodes metastases were diagnosed, then he received bone palliative radiotherapy.

In May 2013 carboplatin and zoledronic acid were given, but a progressive disease was observed in October 2013 (lung, subcutaneous metastases). A biopsy of skin lesions confirmed breast cancer metastases. We commenced eribuline for a total of six cycles with no clinical benefit. Consequently, we interrupted therapy for PD with pulmonary lymphangitis, pulmonary thromboembolism and significant deterioration of the patient’s clinical condition (Eastern Cooperative Oncology Group Performance Status, ECOG PS 2).

**Fig. 1:**
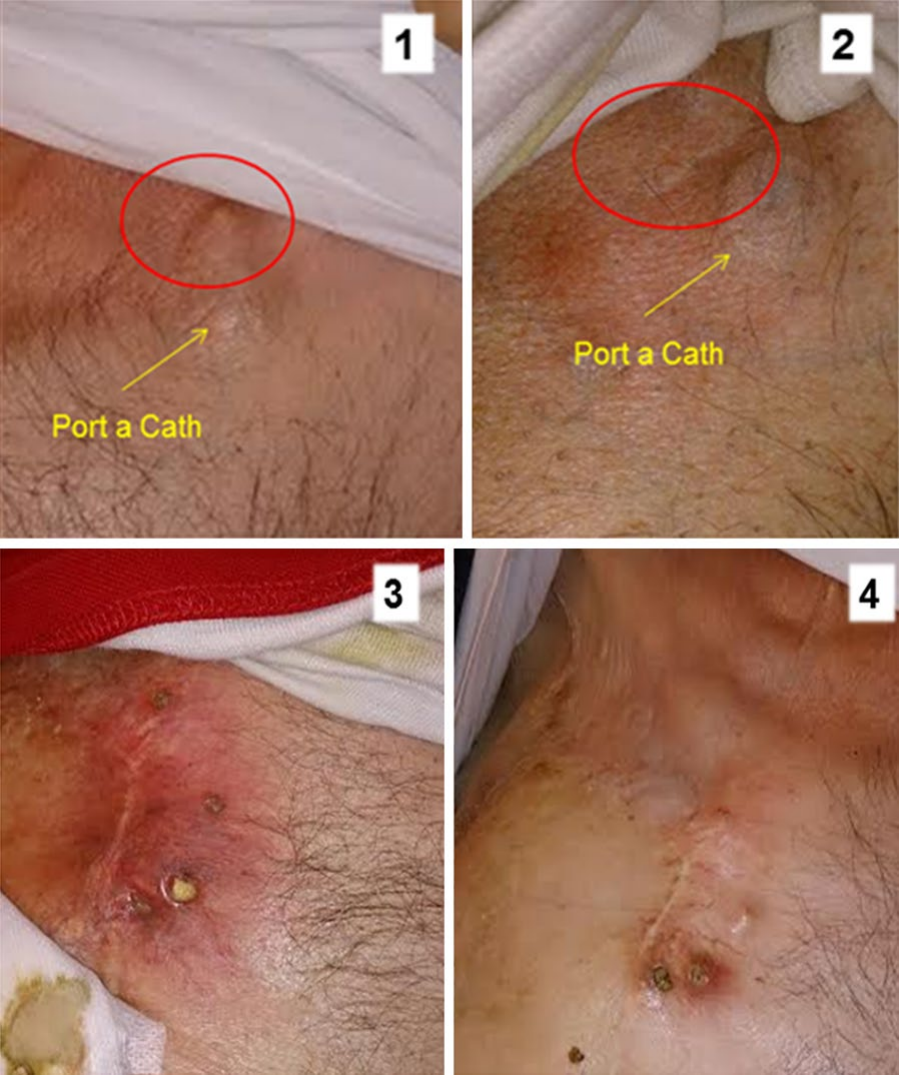
*1.1*, *1.2* Complete response of subcutaneous lesion near the port –à-cath. *1.3*, *1.4* Partial response of ulcerative skin lesion which eventually healed

In May 2014 an off-label treatment with everolimus (EVE) and exemestane (EXE) was requested and approved by Ethics Committee of the Marche Region (CERM). After one month of treatment with everolimus, it was possible to highlight the complete response of the skin lesions had almost completely disappeared and were no longer palpable on physical examination (Fig. 1, Panels 1.1, 1.2). Whereas, a partial response of the other skin lesions located in the right mammary region is evident. At the start of the combined treatment, there were ulcerative lesions which healed (Fig. 1, Panels 1.3, 1.4).

**Fig. 2:**
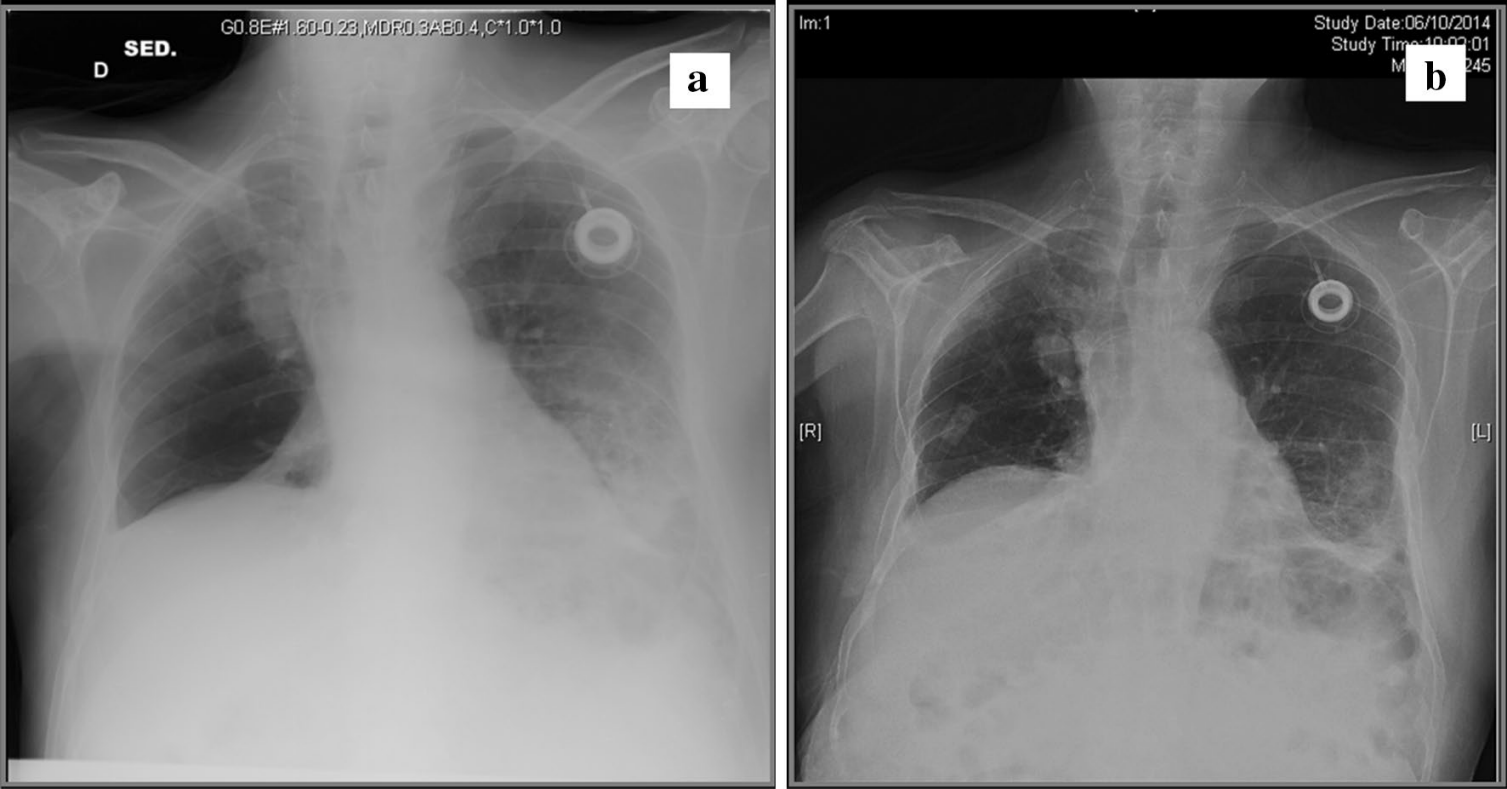
**a** At the start of the fifth cycle dispnea was evident. A chest radiography was done which was strongly suggestive of pneumonitis then we interrupted treatment with everolimus exemestane and we started him on systemic therapy steroid, bronchodilators and supplemental oxygen. **b** A new chest radiography was taken 10 days after cessation of treatment and highlights an improvement in the pneumonitis

Unfortunately, this treatment lasted for 4 months only due to the deterioration of the patient’s clinical condition. Dyspnea was, in fact, evident, at the start of the fifth cycle. The patient complained of coughing and further worsening of his ECOG PS. A chest radiography was done which was strongly suggestive of pneumonitis (Fig. 2a) then we interrupted treatment with everolimus and exemestane and we started him on systemic steroid therapy, bronchodilators and supplemental oxygen. A new X-ray was done 10 days after cessation of treatment and highlights an improvement in the pneumonitis (Fig. 2b).

Despite this improvement in his breathing symptoms, his clinical condition worsened. He became even more cachectic and sadly expired a month later. Table 1 resumed patient’s medical history.

**Table 1:** Patient medical history

Treatment line	Start and stop, year/month	Sites of metastasis	Therapeutic schedule	Best response	TTP (months)
1st	1996/ to 1997/3	Lung	Paclitaxel 80 mg/m2 weekly (day 1–8–15, w4)	PR	12
2nd	1997/4 to 2000/1	Lung	Megestrol 160 mg daily^a^	PR	33
3rd	2000/1 to 2001/1	Lung	Exemestane 25 mg daily	SD	12
4th	2001/2 to 2001/6	Lung	Epirubicin (75 mg/mq day1) and Docetaxel (80 mg/mq day1) w3^a^	SD	4
5th	2001/7 to 2002/1	Lung	Letrozole (2.5 mg daily) and LHRHa	PD	6
6th	2002/2 to 2002/12	Lung	Vinorelbine 25 mg/m2 IV day 1–>5 w3	PD	10
7th	2002/12 to 2007/10	Lung	Megestrol 160 mg daily^b^	PR	58
8th	2007/10 to 2008/5	Lung	Tamoxifen 20 mg daily	PR	7
	2008/6 to 2009/1		No therapy		
9th	2009/1 to 2011/2	Lung	Capecitabine 1000 mg/mq b.i.d. from days 1 to 14, w3	SD	25
10th	2011/2 to 2013/3	Lung	Cyclophosphamide 50 mg/die PO and Methotrexate 2, 5 mg PO twice a week	SD	25
	2013/4	Lung, bone	Palliative radiant treatment for bone metastases of sacral spine.		
11th	2013/5 to 2013/10	Lung, bone, nodes	Carboplatinum (AUC5) w3, Zoledronic acid	PD	5
12th	2013/10 to 2014/4	Lung, bone, nodes, subcutaneous	Eribuline 1.2 mg/m2 day 1–8 w3^c^ Zoledronic Acid	PD	6
13th	2014/5 to 2014/9	Lung, bone, nodes, subcutaneous	Everolimus 10 (5) mg daily and Exemestane 25 mg daily^d^	PR	4

*TTP* time to progression, *PR* partial response, *SD* stationarity of disease, *PD* progression of disease, *LHRHa* Luteinizing hormone releasing hormone (LHRH) agonist

^a^Poor tolerance

^b^Rechallenge

^c^Pulmunary thromboembolism

^d^Deterioration of clinical condition

In 1996, the patient underwent genetic counseling and genetic testing which was negative for BRCA1 and BRCA2 gene mutation.

## Conclusions

Although the hormonal environment in male patients differs from that in female patients, AIs may play a key role in the treatment of male BC patients since in men, 80% of circulating estrogens derived from peripheral aromatization of testicular and adrenal androgens, with direct production from the testes accounting for the remaining 20%. Several studies carried out in healthy men have demonstrated that administration of non-steroidal AIs causes a significant decrease in plasma estrogen. However, data about the impact of AIs on estrogen plasma levels in MBC patients and their clinical efficacy are still lacking [10–14]. Recently, Doyen et al. demonstrated that aromatase inhibition leads to a significant decrease of estrogen level in MBC patients [16]. However, baseline estrogen levels are higher in men than in postmenopausal women because of a higher level of peripheral androgens and AIs do not seem able to inhibit the testicular production of estrogen, which account for 20% of the circulating estrogen. In healthy men, AIs caused a significant increase of LH and FSH [17–19]. These results lead to hypothesize that suppression of estrogen production in male patients by monotherapy using AIs may be suboptimal respect to the combination of AIs with the analogue LH–releasing hormone [16, 20]. Aromatase inhibitors are widely used for treating metastatic male breast cancer patients but, in this setting, their use is not substantiated by prospective clinical trials, rather driven by supposed similarities existing with breast cancer in postmenopausal women [21].

BOLERO-2 trial results demonstrated that the combination of everolimus and exemestane increases the efficacy compared with exemestane in terms of progression-free survival (4–6 months) in a patient population of postmenopausal, hormone receptor positive, advanced breast cancer patients up to one prior chemotherapy treatment [15]. To the best of our knowledge, this is the second case of male breast cancer treated with everolimus. Recently, Kattan J reported the first case of a 74-year-old man who had relapsed after 4 years of adjuvant tamoxifen therapy. Eight subsequent cycles of vinorelbine and capecitabine produced a moderate partial response. Tamoxifen was reintroduced to maintain the outcome, whereas in an attempt to reverse hormone-resistance, tamoxifen and everolimus were combined [22].

Although heavily pretreated, our patient responded very well to endocrine therapy so, despite his poor clinical condition, we proposed a new endocrine line with exemestane and everolimus as an off label treatment ruling out the option of tamoxifen everolimus combination, because of his recent pulmonary thromboembolism.

Besides, today, it’s also uncertain if exemestane is the optimal treatment to adding with everolimus. Ongoing trials have been evaluated the role of everolimus to reverse hormone-resistance in combination with different endocrine agents as ER antagonist (e.g., Fulvestrant) or as monotherapy. On this basis, we are waiting for results of a phase II trial to evaluate the efficacy of everolimus as monotherapy in the treatment of women with ER+ metastatic BC [23].

In our experience, that is the case of a highly pretreated patient with very good response to endocrine therapy and who precociously stopped the everolimus plus exemestane treatment because of the development of pneumonitis. It has already been documented that about 15–20% of female BC patients treated with everolimus and exemestane combination have non-infectious pneumonitis. From BOLERO-2 trial results, approximately one-quarter of events (grade ≥2) occurred within the first 12 weeks (cumulative risk, 5%). Cumulative risks of pneumonitis (grade ≥2) in the EVE + EXE arm were 10 and 16% at 24 and 48 weeks, respectively. Among patients with grade 3 pneumonitis in the EVE + EXE arm, 80% experienced resolution to grade ≤1, typically following dose interruption/reduction, after a median of 3.8 weeks and in case administering systemic steroids, and supplemental oxygen if patient’s symptoms are moderate to severe [24–26]. The pulmonary toxicities incidence in patients affected by BC are similar to those affected by metastatic renal cell carcinoma and pancreatic neuroendocrine tumors which are treated with everolimus respectively in 2nd/3th line and in case of unresectable, locally advanced or metastatic disease [26].

That is the case of long survival male BC patient with luminal B subtype and no BRCA mutations. Furthermore, our patient achieved higher PFS with endocrine therapy than chemotherapy creating the rationale for last line of treatment with everolimus and exemestane combination. He immediately reported an important clinical benefit, as it could be highlighted by the resolution of skin lesions after one month of treatment. Exemestane and everolimus combination offered a partial response with the severe adverse event pneumonitis leading to treatment interruption shortly before the patient expired.

Attending conclusive results from ongoing studies, the combination should not be used routinely in male metastatic BC patients. It might be taking into account for highly selected cases, as male patients with HR positive BC with good response and long time to progression to preceding endocrine lines and especially with very good performance status since the possible severe adverse events.

## Abbreviations

BC: breast cancer
MBC: male breast cancer
AIs: aromatase inhibitors
CT: computed tomography
TTP: time to progression
PR: partial response
SD: stationarity of disease
PD: progression of disease
LHRHa: luteinizing hormone releasing hormone (LHRH) agonist
ECOG-PS: Eastern Cooperative Oncology Group Performance Status
CERM: Ethics Committee of the Marche region
BRCA: BReast CAncer gene
LH: luteinizing hormone
FSH: follicle-stimulating hormone
EVE: everolimus
EXE: exemestane
